# Advanced Spintronic and Electronic Nanomaterials

**DOI:** 10.3390/nano14131139

**Published:** 2024-07-02

**Authors:** Gang Xiang, Hongtao Ren

**Affiliations:** 1 College of Physics, Sichuan University, Chengdu 610064, China; 2 School of Materials Science and Engineering, Liaocheng University, Liaocheng 252059, China

Since single-layer graphene [1] with ultrahigh carrier mobility was obtained experimentally in 2004, two-dimensional (2D) layered electronic materials have become more widespread [2,3,4,5,6,7,8,9]. Two-dimensional non-layered materials [10,11,12,13,14], with their abundant terrestrial resources and low costs, support broader practical applications. Consequently, the repository of 2D materials has become more diverse, facilitating their application in spintronics [15,16,17,18,19], flexible electronics [20,21], information science [22,23], and related fields [24,25].

Over the past two decades, spintronics and electronics [26,27,28,29,30] have developed very rapidly. In 2017, low-temperature long-range ferromagnetic order was experimentally discovered both in Cr_2_Ge_2_Te_6_ [26] and Crl_3_ [27] monolayer systems. Two-dimensional ferromagnetism immediately became of tremendous interest to researchers all over the world. As such, studies on 2D materials have expanded and now correlate with investigations of both traditional materials and emerging materials including diluted magnetic semiconductors and wide band gap semiconductors.

This Special Issue brings together ten articles, specifically eight research articles and two review articles, dedicated to advanced spintronic and electronic nanomaterials. The content of the Special Issue includes the following: the modulation of vortex resonance in ferromagnetic permalloy dots [31], the capping layer effect on tunneling magnetoresistance in tunnel junctions [32], the size-dependent superconducting properties of indium nanowires [33], the co-doping effect of Mn and halogen elements on GeSe monolayers [34], the colossal magnetoresistance in layered diluted magnetic semiconductor Rb(Zn,Li,Mn)_4_As_3_ [35], charge density wave transitions in 2D 1T-TaS_2_ crystals [36], characterizations of Mn_5_Ge_3_ contacts on Ge/SiGe heterostructures [37] and Ni-doped Cd_3_As_2_ films on GaAs (111) substrates [38], strain engineering of intrinsic ferromagnetism in 2D van der Waals materials [6], and spintronic applications of carbon-based nanomaterials [39]. Our Special Issue may promote and accelerate ongoing research efforts of advanced spintronic and electronic nanomaterials. It is of vital importance to 2D spintronic devices and will be of interest to general readers of Nanomaterials.
